# Cytoskeletal Proteins in Cancer and Intracellular Stress: A Therapeutic Perspective

**DOI:** 10.3390/cancers12010238

**Published:** 2020-01-18

**Authors:** Mei Shan Ong, Shuo Deng, Clarissa Esmeralda Halim, Wanpei Cai, Tuan Zea Tan, Ruby Yun-Ju Huang, Gautam Sethi, Shing Chuan Hooi, Alan Prem Kumar, Celestial T. Yap

**Affiliations:** 1 Department of Physiology, Yong Loo Lin School of Medicine, National University of Singapore, Singapore 117593, Singapore; e0013225@u.nus.edu (M.S.O.); phsdes@nus.edu.sg (S.D.); phsceh@nus.edu.sg (C.E.H.); 2 Cancer Science Institute of Singapore, National University of Singapore, Singapore 117599, Singaporecsittz@nus.edu.sg (T.Z.T.); rubyhuang@ntu.edu.tw (R.Y.-J.H.); 3 Department of Pharmacology, Yong Loo Lin School of Medicine, National University of Singapore, Singapore 117600, Singapore; phcgs@nus.edu.sg; 4 School of Medicine, College of Medicine, National Taiwan University, No. 1 Ren Ai Road Sec. 1, Taipei City 10617, Taiwan; 5 Department of Obstetrics and Gynaecology, National University Hospital, National University Health System, Singapore 119074, Singapore; 6 Medical Science Cluster, Cancer Program, Yong Loo Lin School of Medicine, National University of Singapore, Singapore 117597, Singapore; 7 National University Cancer Institute, National University Health System, Singapore 119074, Singapore

**Keywords:** cytoskeletal molecules, mitochondrial stress, endoplasmic reticulum stress, oxidative stress, cancer, therapeutics

## Abstract

Cytoskeletal proteins, which consist of different sub-families of proteins including microtubules, actin and intermediate filaments, are essential for survival and cellular processes in both normal as well as cancer cells. However, in cancer cells, these mechanisms can be altered to promote tumour development and progression, whereby the functions of cytoskeletal proteins are co-opted to facilitate increased migrative and invasive capabilities, proliferation, as well as resistance to cellular and environmental stresses. Herein, we discuss the cytoskeletal responses to important intracellular stresses (such as mitochondrial, endoplasmic reticulum and oxidative stresses), and delineate the consequences of these responses, including effects on oncogenic signalling. In addition, we elaborate how the cytoskeleton and its associated molecules present themselves as therapeutic targets. The potential and limitations of targeting new classes of cytoskeletal proteins are also explored, in the context of developing novel strategies that impact cancer progression.

## 1. Introduction

The cytoskeletal proteins within the cell act in a coordinated manner to enable the proper functioning of all cellular and biochemical processes by regulating the cellular structure, organisation, trafficking and motility. During malignant transformation, the cytoskeletal network can be reprogrammed to aid in the progression of cancer through the promotion of tumour cell survival, growth and invasion, resulting in the tumour cells acquiring the various hallmarks of cancer. Moreover, within the tumour microenvironment, tumour cells are also subjected to various types of cellular and environmental stresses including oxidative stress, endoplasmic reticulum-related proteotoxic stress and mitochondrial stress. The changes that occur during malignant transformation equip the tumour cells with the ability to exploit the cellular stresses to induce increased tumour aggressiveness. This response is facilitated in part by the reorganised cytoskeletal proteins and their associated signalling pathways in the tumour cells. However, the roles that cytoskeletal proteins play in conjunction with the various intracellular stresses are not widely discussed. Additionally, therapeutic approaches targeting the diverse cytoskeletal molecules remain largely unexplored, despite the continuous improvements. In this current review, we will summarise the interactions between the cytoskeletal molecules and several important intracellular stresses, namely oxidative, mitochondrial and endoplasmic reticulum stresses, in both normal and cancer cells. We will also highlight the therapeutic potential of several pre-clinical cytoskeletal drugs, which may become clinically useful strategies in novel cancer therapies.

### 1.1. Cytoskeletal Molecules in Cancer

**Figure 1:**
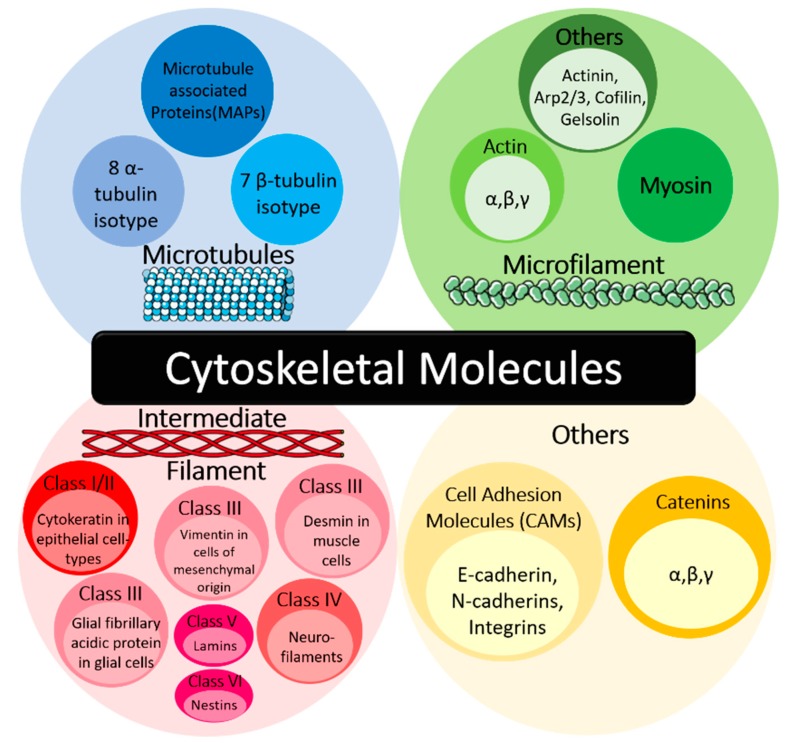
Classification of cytoskeletal molecules. The cytoskeletal molecules can be classified into three main classes, namely the microtubules, microfilaments and intermediate filaments. Within each main class of molecules, it can be further categorised based on its function and molecular type. In addition, there are also other cytoskeletal molecules that do not fall into the above groups, such as the cell adhesion molecules (CAMs) and catenin. Arp, actin-related proteins.

The cytoskeleton is integral to numerous cellular processes and mechanisms, including the spatial organisation of cell content, cellular anchorage to the external environment, regulation of cell morphology and motility, as well as the transportation of intracellular cargo [1]. It consists of three main classes, namely the microtubules, microfilaments and intermediate filaments, which are assembled into networks to carry out their specific, but integrated functions (Figure 1). Under normal physiological conditions, the cytoskeletal network in the cell is resistant to deformation. However, in malignant cells, reorganisation of the cytoskeleton can occur. These modifications in arrangement and composition of the cytoskeleton during transformation involve different cytoskeletons and their associated molecules, such as microtubules and microtubule-associated proteins (MAPs), microfilaments and actin stress fibres [2].

Microtubules are hollow cylindrical structures comprising of α- and β-tubulin heterodimers, of which there are eight α-tubulin and seven β-tubulin isotypes [3]. Microtubules play critical roles in the maintenance of cell shape, trafficking of proteins and organelles, as well as chromosomal segregation during cell division [3]. In tumour cells, differences in the expression of tubulin isotypes and MAPs compared to the normal cells can contribute to disease progression and chemoresistance. The upregulation of βIII-tubulin is associated with tumour aggressiveness and poor prognosis of various epithelial cancers [3]. Differential expression of MAPs, such as increased tau level and downregulation of MAP2c, also confer chemotherapeutic drugs resistance in tumour cells [4,5].

Microfilaments are made up of actin, which consists of three isoforms—α-, β-, and γ-actin [6]. Actin can exist as the globular monomer, G-actin, or the filamentous polymer, F-actin. In normal cells, actin polymerisation and depolymerisation are tightly regulated to facilitate maintenance of cell morphology, adhesion, motility, exocytosis and endocytosis. However, the disorganisation of the actin cytoskeleton during tumorigenesis leads to an alteration of the nuclear:cytoplasmic ratio in cells, as well as promotes tumour formation, survival and metastasis [6]. Cancer cells also have an increased ratio of G:F actin compared to normal cells and the alteration of G:F actin ratios could promote cellular metastasis, which can also be regulated by several signalling proteins, such as Yes-associated protein (YAP) [6,7]. Actin filaments cross-linked by α-actinin can also interact with myosin to form actomyosin bundles called actin stress fibres. They are vital for cell adhesion to the extracellular matrix (ECM), and becomes less pronounced in tumour cells to stimulate their migration [8].

Intermediate filaments are cell-type specific and can be cross-linked to each other or to microtubules and microfilaments [1]. They are subdivided into six classes, including cytokeratin in epithelial cell-types (Class I/II), vimentin in cells of mesenchymal origin, desmin in muscle cells, glial fibrillary acidic protein in glial cells (Class III), neurofilaments (Class IV), nuclear lamins (Class V) and nestins (Class VI) [9,10]. They are usually assembled in response to mechanical stress and contribute to the mechanical integrity of the cells as well as regulation of the cellular space [1,11]. Typically, cancer cells maintain the cell-type specific expression of their intermediate filaments, including that of metastatic tumours [11]. However, in several cancers, such as breast cancer and melanoma, intermediate filaments including cytokeratin and vimentin are co-expressed together in the tumour cells [12]. Reorganisation of the intermediate filaments in tumour cells may result in epithelial–mesenchymal transition (EMT), which promotes cell migration and invasion, resulting in a more aggressive phenotype [13]. For example, several studies have shown that epithelial cancers with an increased expression of vimentin have poorer prognosis due to an augmentation of cell growth as well as EMT, leading to increased cell motility that can give rise to metastasis [14,15,16].

Other types of cytoskeletal proteins are also involved in the cancer pathology, including cell adhesion molecules (CAMs) such as E-cadherin and its associated proteins (e.g., catenin) (Figure 1). In normal cells, α-, β-, and/or γ-catenin bind to E-cadherin on the cell surface membrane to form cell–cell adhesion junctions [17]. Catenin anchor the E-cadherin molecules to the cellular cytoskeleton. However, in tumour cells, there is a general reduction in the expression of both E-cadherin and other catenin, such as α-catenin and p120 catenin in squamous cell carcinoma, breast, colorectal, prostate, and pancreatic cancer [17,18,19]. This results in the loss of cell–cell adhesion, which stimulates increased cell motility, thus aiding in tumour cell migration, invasion and metastasis [17].

### 1.2. Intracellular Stress in Cancer

Cytoskeletal molecules are essential in normal cellular functions and, in cancer, mutations in these cytoskeletal molecules have been reported. One example would be β-catenin, which is highly mutated in liver, colon and prostate cancers, was previously reported to likely promote tumorigenesis through Wnt-dependent activity [20,21]. Alternatively, mutations in the cytoskeletal protein-related coding regions (CPCRs) within the genome and cytoskeletal-related proteins have been reported in several cancers, such as breast, melanoma and ovarian cancers [22,23,24]. These mutations could contribute to altered cytoskeletal activity during carcinogenesis. For example, missense and loss-of-function mutations of filamin A inhibit the disassembly of focal adhesion sites, thereby promoting tumour cell migration and invasion [25]. Apart from having crucial roles in carcinogenesis, cytoskeletal proteins are also closely implicated in cellular stress responses.

Dynamin-related GTPases, optic atrophy type 1 (Opa1) and dynamin related protein 1 (Drp1), are fundamental players in mitochondrial fusion and fission, which are important processes in the stress response of mitochondria [26]. Mitochondrial stress arises from mitochondria dysfunction, which can be caused by multiple factors including loss of membrane potential and reactive oxygen species (ROS) production [26]. Dysregulation of the mitochondrion results in disrupted metabolism and triggers mitochondrial stress response, thus inducing changes in the rate of mitochondrial fusion and fission, biogenesis and mitophagy. Furthermore, the remodelling of the mitochondrial network through fusion and fission in tumour cells can promote proliferation and survival by inhibiting apoptosis [26].

Tubulin isotypes, βIII, βV and βVI, can function as redox switches and oxidative stress sensors. The imbalance in the equilibrium between pro-oxidant and anti-oxidant species due to increased ROS production from the mitochondria enzymatic reactions, peroxisomes and endoplasmic reticulum (ER) could result in oxidative stress [27,28,29,30,31]. Oxidative stress can be carcinogenic as ROS induces damages to multiple molecules including DNA, lipids and proteins in the cells [30]. It also promotes cancer progression by inducing tumour cell migration and invasion through epithelial–mesenchymal transition (EMT), thereby promoting tumour metastasis [30,32].

Moreover, tubulins are also known to be involved in the responses for ER stress. βIII tubulin has been shown to interact with glucose-regulated protein 78 (GRP78), a key molecule in the unfolded protein response (UPR) of the ER, thus demonstrating the significance of microtubules in cellular stress response of cancer cells [3]. ER stress can arise from the accumulation of misfolded and unfolded proteins in the ER, which contributes to proteotoxicity in the cell [33]. Tumour cells are more prone to ER stress with their high proliferation rates and exposure to conditions in the tumour microenvironment, such as hypoxia, thus increasing the susceptibility of misfolded proteins accumulation [34,35]. In response to ER stress, tumour cells exploit the UPR to adapt and confer increased malignancy, for example, by inhibiting apoptosis, inducing angiogenesis and augmenting invasiveness of the tumour cells [34,35]. In the following sections, the roles of cytoskeletal proteins in the various types of cell stresses and their potential for targeted cancer therapies will be discussed in depth.

## 2. The “Cytoskeletons” in Oxidative Stress

Oxidative stress can contribute to the accumulation of DNA damage, which can increase the acquisition of mutations and the activation of signalling pathways, such as extracellular-signal-regulated kinase (ERK), nuclear factor kappa-light-chain-enhancer of activated B cells (NF-κB) and phosphatidylinositol 3-kinase/protein kinase B (PI3/AKT) and reactive oxygen species (ROS)-associated epithelial–mesenchymal transition (EMT), thus promoting tumour development in several cancers including that of the breast, stomach and liver [28,30,36,37,38]. Moreover, the interaction of the cancer cells and inflammatory response in the tumour microenvironment also contribute to persistent ROS production and tumour progression [28,30]. Different classes of cytoskeletal proteins are known to affect oxidative stress. Actin and its associated proteins, such as gelsolin (GSN) and Ras homolog family member A (RhoA), can regulate oxidative stress by affecting the level of intracellular ROS. These proteins also serve as oxidative stress adaptor, responding to oxidative stress to induce changes in cytoskeletal organisation and downstream cellular effects, such as cellular survival, motility, migration and invasion.

### 2.1. Actin and Its Regulators in Oxidative Stress

Firstly, the interaction between the Rho/Rho-associated protein kinase (ROCK)-driven actomyosin contractibility and oxidative stress can result in the suppression of cellular migrative capability of cells. In melanoma, oxidative stress can be enhanced with the increase in intracellular ROS levels via a Rac1-dependent mechanism when actomyosin contractibility is reduced [39,40]. This can lead to activation of oxidative stress response, where p53-inducible gene 3 (PIG3), which is regulated by tumour protein p53 (TP53) transcription, are upregulated during ROS-induced damage response. This, in turn, promotes the activation of Rho GTPase activating protein 5 (ARHGAP5) and further inhibits actomyosin contractility via the inhibition of ROCK/Rho/myosin light chain 2 (MLC2) signalling. The inhibition of ROCK/Rho/MLC2 signalling then results in the suppression of migratory capability of cells [39,40]. The feedback loop between actomyosin contractibility and the TP53–PIG3 transcriptional axis in ROS management to suppress cell motility suggests a potential in cancer therapeutics through the inhibition of actomyosin contractibility by targeting Rho kinases [39,40].

Apart from actomyosin network, other actin regulators also play important roles in regulating ROS. GSN, an actin regulatory protein involved in the severing and capping of actin, enhances the invasive capabilities of colorectal cancer cells by increasing intracellular superoxide levels and secretion of urokinase plasminogen activator (uPA), which promotes matrix degradation and tumour invasion [41,42,43]. The increase in superoxide level was attributed to the inhibition of the enzymatic activity of copper- and zinc-containing superoxide dismutase (Cu/ZnSOD) by gelsolin [42]. On the contrary, Ras homolog family member A (RhoA) can disrupt actin organisation, which would likely affect cellular motility and function [44]. In the presence of intracellular ROS, RhoA undergoes proteolysis under stress to form stable amino- and carboxyl-terminal RhoA fragments. The presence of both fragments results in the disruption of the actin stress fibre organisation, whereby formation of disorganised stress fibres and assembly of nuclear actin rods were observed [44]. Actin stress fibre formation is essential in cellular adhesion, migration and actomyosin contractibility [8] and, hence, its disorganisation may suggest a potential effect on cytoskeletal remodelling and altered motile capacity. On the other hand, the formation of nuclear actin rods could have a protective role to rescue the cell during cellular stress, possibly similar to how cofilin mediates actin rod formation during stress [45].

Functional actin also has an important protective role in cellular stress. It was reported that a cysteine-to-alanine mutation at actin residues 284 and 373 resulted in the reduction in oxidised actin bodies (OABs) formation and the reduced rate of cell recovery under oxidative stress [46]. OABs are stable structures formed upon the oxidation of actin and it has been suggested that actin and its associated proteins are sequestered within OABs and thus protected from oxidative damage. The temporary loss of actin remodelling prevents growth, endocytosis and allows for cells to recover. Hence, the presence of normal actin may serve as an adaptive response during oxidative stress, as observed in yeast studies [46]. However, the formation and function of these OABs in mammalian cells require further investigations.

### 2.2. Other Cytoskeletal Proteins in Oxidative Stress

Cytoskeletal proteins of the intermediate filament class, such as vimentin, have also been reported to be upregulated significantly in liver tumours induced by oxidative stress compared to normal liver in a rat model [47]. This was also observed in human liver cancer whereby IQ motif containing GTPase activating protein 1 (IQGAP1) and vimentin were upregulated [47]. These suggest a possible correlation between cytoskeletal protein upregulation and oxidative stress.

**Figure 2:**
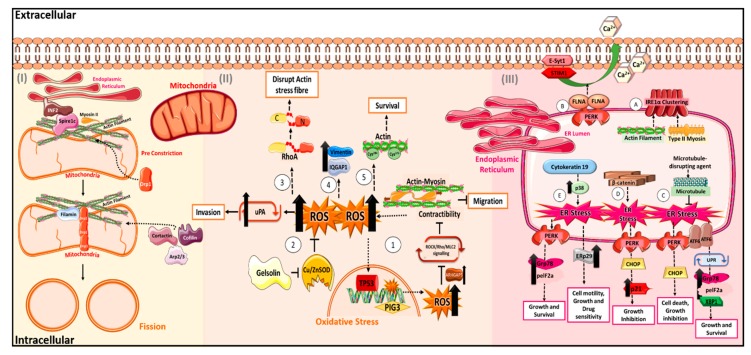
Overview of how some cytoskeletal molecules function in cellular stress. (**I**) Cytoskeletal proteins facilitate the Drp1-mediated mitochondria fission through the recruitment of Drp1 to the contact sites and subsequent fission with the aid of translocated cofilin, Arp2/3 and cortactin to the mitochondria. (**II**) Cytoskeleton and its associated proteins can function in oxidative stress. Oxidative stress can be regulated by actin and its associated proteins such as myosin and gelsolin to affect migration and invasion (1,2). On the other hand, oxidative stress acts to promote survival, actin stress fibre disruption and changes in gene expression (3,4,5). (**III**) Cytoskeletal molecules are important in the colocalization and function of important ER sensors in the presence of ER stress (A,B). In addition, other cytoskeletal molecules such as microtubules and β-catenin can activate downstream ER response pathways to promote cell death, growth inhibition and drug resistance. Cytokeratin 19 can also promote ER stress and upregulation of ER proteins to induce changes in cellular behaviour (C,D,E). Arp, Actin related protein; ATF, Activating transcription factor; Bip, Binding immunoglobulin protein; Ca2+, Calcium ion; CHOP, C/EBP homologous protein; Cu/ZnSOD, Copper- and zinc-containing superoxide dismutase; Drp, Dynamin-related protein; eIF2, Eukaryotic Initiation Factor 2; ER, Endoplasmic reticulum; ERp, Endoplasmic reticulum protein; E-Sty1, extended synaptotagmin-1; FLNA, Filamin A; GRP, Glucose-Regulated Protein; IQGAP1, Ras GTPase-activating-like protein IQGAP1; INF, Inverted Formin; IRE, Inositol-requiring enzyme; MLC2, Myosin light chain 2; PERK, Protein kinase RNA-like endoplasmic reticulum kinase; PIG, p53-inducible gene; ROS, Reactive oxygen species; ROCK, Rho-associated protein kinase; Rho, Ras Homolog; RhoA, Ras Homolog, Ras homolog family member A; SPIRE1, Protein spire homolog 1; STIM1, stromal interaction molecule 1; Tm, tropomyosin; TP53, Tumour protein p53; UPR, Unfolded protein response; XBP, X-box binding protein.

Thus, the interplay between the cytoskeleton and pathways that regulate oxidative stress are important for maintenance of cellular homeostasis. Dysregulation of this interplay could contribute to the changes in cancer cell behaviour, like motility and migration, as seen in how actomyosin contractibility and gelsolin activity affect cellular motility through its effects on intracellular ROS (Figure 2).

## 3. The “Cytoskeletons” in Mitochondrial Stress

### 3.1. Drp1 and Actin in Mitochondrial Fission and Fusion

As the energy powerhouse, the homeostasis and proper regulation of the stress responses of mitochondria are vital processes for cells [48]. In mammalian cells, mitochondria do not remain static, but rather constantly change in response to other signals such as metabolic and environmental stresses [48]. One of the significant features of mitochondrial dynamics is the balance between mitochondrial fission and fusion [48]. Generally, fission of existing mitochondrial network generates new mitochondria, which can be an efficient way to facilitate the degradation of damaged mitochondria, and fusion of partially damaged mitochondria to the network could help to rescue mitochondria from the stresses [48]. Disrupted mitochondrial fission and fusion not only affect normal cellular functions but are also linked to the pathogenic conditions such as neurodegenerative diseases and cancer [48].

A group of GTPases has been identified to regulate the fission and fusion process of mitochondria. Importantly, fusion regulators include Mitofusin 1 and Mitofusin 2, which control the fusion of the mitochondrial outer membrane, and optic atrophy type 1 (Opa1), which regulates inner membrane fusion [48,49]. Dynamin related protein 1 (Drp1) has been discovered to drive the mitochondrial fission on both inner and outer membranes. The Drp1-mediated mitochondrial fission is proposed to be initiated by the endoplasmic reticulum (ER), where the ER forms a close contact with mitochondria at contact sites known as mitochondria-associated ER membranes (MAMs) [50]. ER tubules twist around mitochondria, inducing a pre-constriction event that decreases the mitochondrial cross-sectional diameter and allows for Drp1 assembly at the site of fission (Figure 2).

Recent studies have suggested that actin polymerisation at the ER–mitochondria interface may be a key component in the pre-constriction process for Drp1-dependent mitochondrial fission. Inverted formin 2 (INF2), which regulates both actin polymerisation and depolymerisation processes, localises to ER and remodels actin cytoskeleton, which subsequently recruit Drp1 for fission to happen [51]. Spire1C, an actin-nucleating protein, localises to mitochondria and binds INF2 to promote actin assembly on mitochondria surfaces [52]. The local polymerisation of actin induced by these factors is important for the subsequent recruitment and oligomerisation of Drp1 at mitochondria, as actin filaments can bind Drp1 directly to increase its GTPase activity, which is essential for mitochondrial constriction and fission [53]. It is further found that the interaction between actin and Drp1 is bridged via the scaffold protein filamin [54]. Besides actin polymerisation factors, other actin regulators with depolymerising or branching functions have been shown to regulate mitochondrial fission. Cofilin, actin related protein 2/actin related protein 3 (Arp2/3) and cortactin translocate to mitochondria after treating with mitochondria-damaging agents, and depletion of these proteins leads to elongation of mitochondria and abnormal mitochondrial network [53]. Myosin II, the actin motor protein, is recruited to mitochondria under the regulation of INF-2, and is required for the mitochondrial Drp1 localisation [55] (Figure 2).

In addition to the Drp1-depedent mitochondrial fission, the INF2–Actin axis could induce the fission of mitochondrial inner membrane via increasing mitochondrial matrix calcium [56]. Actin increases the contact between mitochondria and ER, where the calcium is released to mitochondria from ER. Elevated mitochondrial calcium in turn activates constriction of mitochondrial inner membrane, in a myosin IIA-dependent and Drp1-independent manner [56].

Unlike fission, the roles of the actin cytoskeleton in mitochondrial fusion are less characterised. It is currently thought that actin dynamics are important for both fission and fusion to maintain the balance [57]. It is observed that actin behaves in a cyclic manner during fission and fusion, with the assembly of actin occurring at the outer membranes of healthy and elongated mitochondria [57]. Upon assembly, mitochondria fission is induced while fusion is inhibited. Following fission, actin disassembles from the fragmented mitochondria, and fusion occurs leading to the integration of fragmented mitochondria back into the network.

As the balance of mitochondrial fission and fusion is important to maintain cellular homeostasis, targeting mitochondrial fission/fusion may be effective strategies for combating diseases like cardiovascular diseases and cancer. Currently, there is no effective way of targeting fission or fusion. Modifying agents affecting the actin cytoskeleton to achieve a more focused effect on mitochondrial fission and fusion may; therefore, be an alternative way to target mitochondria.

### 3.2. Regulation of VDAC by Cytoskeleton

Besides roles in regulating mitochondrial fusion and fission, the cytoskeleton has also been suggested to associate with various mitochondrial proteins to regulate the functions of the mitochondria. Voltage-dependent anion channel (VDAC) is one of the well-studied mitochondrial outer membrane proteins being regulated by cytoskeletal elements [58,59,60,61,62,63,64]. VDAC forms channels on the mitochondrial outer membrane, where the regulated channel opening and closure are critical for controlling the permeability of mitochondrial membrane and the flux of ions and metabolites into the mitochondria [58]. Due to its roles in mitochondria, VDAC regulates multiple cellular processes, such as cell metabolism and apoptosis. It has been shown that both actin and microtubules could participate in the regulation of VDAC. Mitochondrial tubulin, which is located on the mitochondrial membrane, was shown to interact with VDAC [59], via its C-terminal tail [60,61]. The αβ-heterodimer of tubulin interacts with VDAC, and nano-molar concentrations of αβ-heterodimers can cause the closure of VDAC [60]. Regulation of VDAC by tubulin has been linked to altered mitochondrial metabolism and function. In cancer cells, free tubulin predominantly inhibited two isoforms of VDAC, VDAC1 and VDAC2, while VDAC3 is minimally affected [64]. The closure of VDAC by tubulin leads to decreased mitochondrial membrane potential of cancer cells, which could eventually alter mitochondrial metabolism to favour the Warburg effect [62,63,64].

The actin cytoskeleton has also been suggested to play a role in VDAC regulation. G-actin directly interacts with VDAC [65]. Moreover, G-actin, but not F-actin, enhances the closure of VDAC in vitro [66]. Gelsolin (GSN), an actin severing and capping factor, has also been shown to bind to VDAC [67]. In response to apoptotic stimuli, GSN inhibits VDAC from opening, thereby preventing the permeabilization of mitochondrial membrane and cytochrome c release, leading to the inhibition of the mitochondria-dependent apoptotic pathway [67].

Taken together, the interplay between the cytoskeleton and mitochondrial dynamics, including ion channel regulation, offer new perspectives in understanding the importance of the cytoskeleton in mitochondrial function as well as the stress responses of the mitochondria.

## 4. The “Cytoskeletons” in Endoplasmic Reticulum Stress

Endoplasmic reticulum (ER) stress is the consequence of the disruption in ER function arising from changes in external and internal stimuli, hence leading to the accumulation and/or aggregation of unfolded or misfolded proteins [68]. In response to ER stress, cells activate either unfolded protein response (UPR)-dependent or independent mechanisms to re-establish the normal ER functionality. Several essential ER sensors, including the pancreatic endoplasmic reticulum kinase (PERK), activating transcription factor 6 (ATF6) and the inositol-requiring enzyme 1α (IRE1α), are activated and can dimerise with itself in the presence of ER stress. This activates the ER stress signalling pathway to reduce ER load and promote cellular survival [69,70,71].

### 4.1. Cytoskeleton in ER Sensors

Cytoskeletal proteins have also been suggested to be involved in the regulation of ER stress and there have been reports of actin and its associated proteins being essential for the clustering and function of ER sensors. The clustering of the ER sensor, IRE1α, in highly ER-stressed cells were abrogated upon treatment with latrunculin-A, an actin-disrupting compound. In addition, the reduction in IRE1α clustering was also observed with the complete loss of function of type II myosin [72]. The loss of nonmuscle myosin II (NMIIB) would inhibit downstream X-box binding protein 1 (XBP1) activity in response to ER stress, indicating its importance for the aggregation of IRE1α [73]. These observations suggest the importance of functional actin filament and myosin in the concerted coordination between the ER and cytoskeleton to activate ER sensors. Activated PERK, another ER sensor, would require the interaction with actin-binding protein filamin A (FLNA) for its downstream activity to promote F-actin network modifications [74]. This allows the relocation of ER associated tethering proteins, stromal interaction molecule 1 (STIM1) and extended synaptotagmin-1 (E-Syt1), to the plasma membrane to overcome the stress induced by Ca^2+^ imbalance [74].

### 4.2. Regulation of ER Signalling Cascade by Cytoskeleton

In addition, cytoskeletal molecules were reported to induce ER stress and its downstream signalling pathway to affect cellular growth, survival, motility and drug sensitivity. Firstly, microtubules were found to be essential in the formation, morphology and dynamic movement of the ER tubules as the ER membrane slides along or attaches to the polymerising microtubules and, in cooperation with molecules motors, can aid in short movement of ER [75,76]. The loss of microtubule function using a novel microtubule disrupting compound, N-deacetyl-N-(chromone-2-carbonyl)-thiocolchicine (TCD), in hepatocellular carcinoma resulted in inhibition of tumour growth and promoted ER stress-induced cell death through the PERK- and ATF6-mediated activation of downstream signalling cascade [77]. In addition, microtubule-targeting agent, taxane, and vinblastine can also activate ER stress-induced UPR. This results in downstream splicing of XBP1, upregulation of glucose-regulated protein 78 (GRP78) and phosphorylation of eukaryotic initiation factor 2α (eIF2α) to promote cell survival [78]. The importance of microtubules in the initiation and regulation of ER stress has been clearly established, as the inhibition of microtubules function can affect ER stress response. Cytokeratin 19, which belongs to the intermediate filament, causes a reduction in cell motility and growth and affect drug resistance in breast cancer through the enhancement of p38 signalling [79,80]. p38 signalling was identified to be important to ER stress as it is interlinked with PERK/p-eIF2α/ATF4/6, PERK/nuclear factor erythroid 2-related factor 2 (NRF2) and XBP-1 [81,82]. The increase in p38 signalling induces ER stress signalling response, which in turn regulates cellular growth and survival through the activation of PERK. Activated PERK would induce the upregulation of downstream Grp78/Bip and phosphorylation of eIF2α. In addition, the p38/XBP-1 pathway can also regulate the expression of endoplasmic reticulum protein 29 (ERp29), contributing to the reduction in cell motility, growth and drug sensitivity against cisplatin and doxorubicin. Hence, the level of cytokeratin 19 in breast cancer cells can likely regulate ER stress induction to promote malignant cell dormancy [79]. β-catenin can activate ER stress and result in growth inhibition in cancer cells upon drug treatment. In myeloma cells, the accumulation of β-catenin occurs with the of phosphorylation of β-catenin by protein kinase C (PKC) with enzastaurin treatment. Accumulated β-catenin activates ER stress, possibly through PERK, and can result in the activation of eIF2α and CCAAT-enhancer-binding protein homologous protein (CHOP). This could cause an upregulation of p21 and subsequent growth inhibition in cells [80].

Collectively, cytoskeletal molecules have been shown to be essential for ER sensor functions and can interact with the ER stress signalling cascades to result in cellular growth inhibition, survival and alteration of drug sensitivity (Figure 2). The multiple roles of cytoskeletal molecules in the ER stress response pathway suggest that these classes of molecules could be potential druggable targets for the treatment of ER-related diseases.

## 5. Effects of Cellular Stress on Chemotherapeutic Response

The interaction between cellular stress response and cytoskeletal molecules have been reported to affect drug sensitivity where the sensitivity of the cancer cells under endoplasmic reticulum (ER) stress towards microtubule-targeting drugs can vary based on the different signalling cascades involved. For example, the loss of glucose-regulated protein 78 (GRP78), which can lead to enhanced ER stress, resulted in the increase of sensitivity towards microtubule-targeting drugs taxol and vincristine, and enhanced cell death in breast cancer through c-Jun N-terminal kinase (JNK) and caspase 7 activity [78]. In contrast, in melanoma cells, ER stress induces drug resistance with the activation of the phosphatidylinositol 3-kinase/protein kinase B (PI3/AKT) pathway through the X-box binding protein 1 (XBP1) protein upon treatment with microtubule-targeting drugs, docetaxel and vincristine [83]. Therefore, the analysis of how cellular stresses interplay with cytoskeletal-targeting agents are important, as these drugs may have different effects depending on the types and stages of cancer. In addition, differential regulation of cytoskeletal proteins within the heterogenous populations during tumour development could possibly affect the systemic coordination that aids in cellular dynamics and biomolecules transportation. This also makes the cytoskeleton as well as its associated proteins and signalling pathways attractive targets for cancer therapy, hence potentially improving cancer mortality [84,85].

## 6. Targeting the Cytoskeleton as Potential Therapeutics

### 6.1. Targeting Microtubules in Cancer

Targeting the cytoskeleton is not a novel strategy in cancer therapeutics; the clinical efficacy of targeting cytoskeleton in cancer has been shown as early as fifty years ago, by their anti-mitotic capabilities [86]. Currently, microtubule-specific chemotherapeutic agents, such as paclitaxel and vinblastine, are the only cytoskeletal-specific poisons used in chemotherapy and remain the mainstay in cancer treatment [87,88]. There are two main classes of microtubule-targeting chemotherapeutics, microtubule polymerisation-inhibiting or microtubule-stabilising agents [87,89]. Examples of microtubule-specific chemotherapeutics include vinca alkaloids, taxanes and epothilones (Table 1) [90]. Taxanes, such as docetaxel and paclitaxel, and epothilones, such as ixabepilone, are examples of successful microtubule stabilising poisons which have achieved clinical utility, by maintaining the structure of the polymerised microtubules [90]. However, microtubule dissociation is often found to be inhibited at high concentrations [91,92]. Despite the diversity of microtubule-binding anticancer chemotherapeutics, the overexpression of different tubulin isotypes and microtubule-interacting proteins that affect drug delivery and metabolism would contribute to drug resistance. For instance, drug resistance against vinca alkaloids can be developed with the increased expression of ATP-binding cassette (ABC) superfamily of transporter proteins, the P-glycoprotein (P-gp) efflux pump [93,94,95,96]. Hence, resistance remains an important unmet clinical problem that calls for the development of effective novel compounds [91,97]. The identification of novel inhibitors, such as IMB5046, with anti-tumour effects in cell lines resistant to existing microtubule inhibitors (such as vincristine and paclitaxel), as well as the discovery of epothilones, provide alternative options of microtubule-specific drugs with better efficacy and lower toxicity. Nevertheless, the current solution to overcoming chemoresistance remains a race between developing new analogues and tumour cells acquiring resistance [5,98,99].

Apart from microtubules and MAPs-specific targets, other members of the cytoskeleton family —actin microfilaments, intermediate filaments, and cytoskeletal-associated proteins and signalling molecules—remain unexplored as therapeutic targets and warrant further investigations for potential clinical utility [88].

### 6.2. Unexplored Therapeutic Targets—Actin Microfilaments

Actin microfilaments have important cellular functions during cancer progression, particularly in cell proliferation and cell migration [85]. Gelsolin (GSN), an actin-associated protein, promotes cell scattering and invasiveness through mediating the phosphatidylinositol 3-kinase/protein kinase B (PI3K/AKT) pathway, reactive oxygen species (ROS) production and secretion of matrix degrading enzymes [41,42,43]. Actin also plays a crucial role in RhoA/Rho-associated protein kinase (ROCK)-mediated cell motility for the migration of cancer cells during metastasis by increasing cellular contractions and motor protein activity that enhances cellular movement [85,100]. Potential actin-specific chemotherapeutics, which had shown promise both in vitro and in vivo include cytochalasin, chaetoglobosins, jasplakinolide, latrunculins and MKT-077 (Table 2) [88,101,102,103,104,105,106,107,108,109,110,111,112,113,114,115,116,117,118,119,120]. These actin-targeting drugs function to promote the depolymerisation of actin, inhibit actin polymerisation or cross-linking actin to disrupt the cellular actin organisation (Table 2) [88,101,102,103,104,105,106,107,108,109,110,111,112,113,114,115,116,117,118,119,120]. However, due to the importance of actin filaments in normal cell physiology, targeting actin microfilaments non-specifically could result in adverse effects such as lethality, as well as cardiac and renal toxicity (Table 1) [88,101,102,103,104,105,106,107,108,109,110,111,112,113,114,115,116,117,118,119,120]. Due to the toxicity in normal cells using conventional actin-disrupting agents, it is imperative to develop new actin toxins with selectivity towards cancer cells. Instead of targeting actin filaments which are found ubiquitously, another possible approach could be to target actin-associated proteins which show distinct differential expression in tumour cells, compared to normal cells [41,43]. In addition, employing effective drug delivery systems with existing actin toxins might be an alternative strategy to achieve good therapeutic windows for treating cancer while maintaining minimal toxicity to normal cells.

### 6.3. Unexplored Therapeutic Targets—Intermediate Filaments 

Intermediate filaments have been shown to exhibit tissue specificity, potentially making them more suitable targets compared to microfilaments in combating tissue-specific malignancies [88]. Potential intermediate filaments specific targets include nestin and vimentin, due to their known involvement in tumorigenesis and metastasis [85,136]. Nestin, a type VI intermediate filament, is often re-expressed in cancer cells, including gliomas, osteosarcoma, colorectal and prostate cancers [137,138,139]. Nestin has been shown to function as a survival factor to inhibit CDK-5 dependent apoptosis, whilst the knockdown of nestin in cancer cells was reported to reduce cell motility [140,141]. On the other hand, vimentin is a type III intermediate filament expressed in diverse cell types, in particular, mesenchymal cells [142]. Overexpression of vimentin has been observed in carcinomas of the breast, lung, gastrointestinal and nervous system, and coincidentally, these cancers are generally associated with greater invasiveness and poorer prognosis [14].

As intermediate filaments may be specifically overexpressed in certain invasive carcinomas but not in normal healthy cells, targeted therapy is possible with minimal side effects and toxicity. For instance, Withaferin-A, a natural bioactive compound, has been shown to induce apoptosis against vimentin-expressing cancer cells [121], while Silibinin can result in the downregulation of vimentin and metalloproteinase-2, thereby reducing prostate cancer cell invasion and motility (Table 1) [124].

### 6.4. Unexplored Therapeutic Targets—Cytoskeletal-Associated Proteins

One class of cytoskeletal-associated proteins which may be potential therapeutic targets, would be the integrins (ITGs) [143]. Liver cancer, melanoma and myeloma were observed to have a significantly higher expression of several integrin genes, suggesting their importance in tumour development [144,145,146,147,148]. Moreover, upregulation of several integrins in multiple cancers including glioblastoma, cervical, lung, liver and pancreatic cancer, also imply their potential to be drug targets for cancer treatment [149]. Altered integrin expression has also been reported in various cancer types [150]. Integrins can form complex synergistic signalling networks with the cancer cells and their surrounding microenvironment, thereby regulating cancer progression [143,151,152,153,154,155]. Integrin signalling can lead to the activation of pathways including nuclear factor kappa-light-chain-enhancer of activated B cells (NF-κB), and increased B-cell lymphoma 2 (Bcl2) expression to promote cell survival and inhibit apoptosis [152,153,154,155]. Several integrins (e.g., αvβ3) also interact with receptors, such as fibroblast growth factor receptor (FGFR) and epidermal growth factor receptor (EGFR), to induce angiogenesis and alter the expression of metalloproteinases to promote cellular invasion [156,157,158]. Integrin antagonists have shown promise in both preclinical and clinical studies, where the cytoskeleton may be indirectly affected due to its close relationship with integrins [151,159,160]. Vitaxin, an integrin antagonist that specifically targets integrin αvβ5, can inhibit angiogenesis through the impairment of the vascular response to endothelial cell growth factors and its signalling pathway such as the extracellular-signal-regulated kinase (ERK) pathway [161,162,163]. Concurrently, the antagonist also prevents the activation of metalloproteinases, hence inhibiting cell motility. Since the start of its development, hundreds of integrin inhibitors have successfully made it into clinical trials, targeting different subunits and isoforms. In particular, antagonists of αvintegrins, which are expressed in many cancers, have shown clinical success with anticancer capabilities [127]. An example of αvintegrin-specific therapeutic in clinical trials is Abituzumab, a humanised, de-immunised monoclonal IgG2 antibody developed by Merck KgaA, which has showed favourable outcomes in castration-resistant cancer patients and metastatic colorectal cancers (Table 1) [128,129,130].

A novel class of anti-cancer compounds in early development, which belongs to the group of cytoskeleton-associated proteins, is isoforms of tropomyosin. In an earlier study, the anti-tropomyosin drug TR100, which targets Tm5NM1/2 by disrupting interactions between tropomyosin and actin, displayed anticancer properties in melanoma and neuroblastoma cells in vitro [132]. Following the discovery of TR100, other anti-tropomyosin analogues have since been developed, such as ATM-3507, which showed enhanced inhibition of tumour growth and increased survival outcome when used in conjunction with anti-microtubule poisons in a mouse xenograft model (Table 1) [133].

The cytoskeletal dynamics of the cell might be potentially targeted to inhibit cell migration and invasion, through targeting actin-binding proteins. One example would be the LIM kinase family of proteins, which are important regulators of actin assembly in the formation of membrane protrusions and cell motility, and play essential roles in metastatic cancer progression [134,164,165]. The overexpression of cofilin (CFL), which is an actin depolymerising protein required for the turnover of actin filaments, enhances cancer cell motility in glioma cells and colorectal cancer cells [134,166,167]. LIM kinases can phosphorylate CFL to suppress actin turnover and stabilise actin filament, thus reducing cell motility [134,135,168,169,170]. However, dephosphorylation of cofilin could also inhibit cell motility in lung cancer [171], suggesting that CFL might act in a cell-type dependent manner. Interestingly, LIM kinase inhibitor, Curcolonol, a sesquiterpene isolated from medical herbs, was reported to reduce breast cancer cell migration [172]. This suppression was suggested to be caused by the reduction in CFL phosphorylation with the inhibition of LIMK1 activity [172]. Moreover, LIM kinases were also recently shown to increase microtubule depolymerisation when overexpressed and regulate microtubule organisation including mitotic spindle assembly [170]. Consistent with this, the inhibition of LIM kinase could hence disrupt mitosis, and act in synergy with microtubule-specific inhibitors to impair mitosis at the G2/M phase [135,173]. This reduces cancer cell proliferation with the initiation of apoptosis [135,173]. Several LIM kinase inhibitors have also been successfully developed, which preferentially target tumour cells in several cancers such as pancreatic, prostate, cervical and glioma carcinomas [135]. LIM kinase inhibitors could; thus, provide improved options for therapeutic success, through the regulation of actin and microtubule functions, preventing migration and invasion in breast cancer as well as proliferation in several other cancers respectively (Table 1).

Finally, cytoskeletons are subjected to post-translational modifications (PTMs), such as deacetylation and detyrosination of tubulin [174,175,176]. Therefore, targeting the PTM, which governs cytoskeletal changes can be an attractive strategy to overcome the side effects of using cytoskeletal toxins (described earlier). For instance, histone deacetylase 6 (HDAC6) has been reported to deacetylate cytoskeletal proteins including α-tubulin and cortactin, thus regulating cytoskeleton dynamics to affect cell migration and metastasis [177]. The HDAC6 inhibitor, Tubacin (tubulin deacetylation inhibitor), has been shown to increase α-tubulin acetylation and reduce cell motility, and has anti-proliferative and pro-apoptotic effects in tumour cells [178]. Moreover, combinatory treatment of HDAC6 inhibitor with proteasome inhibitor resulted in increased cytotoxicity in multiple myeloma [178,179]. Multiple drugs have been reported to affect tubulin acetylation and have exhibited anticancer properties including thiol-based HDAC6 inhibitors [180]. These have been identified to increase tubulin acetylation and exert growth inhibitory effects on colorectal and breast cancer cells [181]. These classes of drugs could potentially work in conjunction with other cancer therapeutic drugs and increase cytotoxicity towards cancer cells.

## 7. Conclusions

In summary, the cytoskeleton and its interacting partners have diverse and critical effects on mitochondrial, endoplasmic reticulum (ER) and oxidative stress related responses. Moreover, multiple aspects of tumour cell behaviour that contribute to tumour progression are affected by various cytoskeletal molecules, including increased cell survival associated with drug resistance, as well as invasiveness associated with high grade cancers and metastasis. Hence, there may be an enormous potential in targeting cytoskeleton therapeutically in cancer treatment, as targeting other cytoskeleton proteins and cytoskeleton-associated proteins still remains unexplored. Microtubule chemotherapeutics have been available for clinical use, albeit with the drawbacks of high toxicity and chemoresistance. New analogues targeting the microtubules are being developed to combat drug resistance, with ongoing clinical trials for several microtubule-targeting drugs, such as iso-fludelone (NCT01379287) and eribulin mesylate (NCT00365157) for several cancers [182,183,184,185]. Other potential therapeutic targets of the cytoskeletons could include microfilaments, intermediate filaments, and cytoskeleton-associated proteins, as summarised in Table 1 and Table 2.

Although current cytoskeleton-targeting drugs inhibit cell proliferation and trigger apoptosis to result in overall shrinkage of tumours, the reduction in tumour size may not necessarily correlate with patient survival, based on studies from different tumour types [186,187,188]. This is possibly due to the heterogeneity of tumours, comprising of multiple subpopulations of cells with distinct genetic profiles [189,190]. For instance, dormant cancer stem cells are capable of escaping the cytotoxic effects of chemotherapeutics with tumour-initiating capabilities, and may develop into new tumours with more aggressive phenotypes [191,192]. Tumour heterogeneity could also affect cytoskeletal protein expression, function and organisation to facilitate cellular dynamics and coordination [149,193,194]. Thus, understanding the specific mechanisms of cellular stress, including the impact of cytoskeletal contributions to stress responses, can facilitate the development of novel targeted therapeutics, directed towards eradicating specific niches in tumours, such as cancer stem cells and invasive subpopulations.

## Figures and Tables

**Table 1:** Examples of drugs targeting other classes of cytoskeletal molecules and cellular effects. Different classes of cytoskeletal proteins can be targeted and have the potential to be targeted. These include well known drugs, such as taxanes and epothilones, that target microtubules and less known drugs that target the intermediate filaments. Moreover, the targeting of cytoskeletal associated proteins can also affect cytoskeletal proteins to bring about cellular changes and inhibit cancer progression.

Targeted Proteins		Drugs	Action and Cellular Effects	Ref.
**Microtubules**		Taxanes (e.g., Docetaxel, Paclitaxel)	Stabilisation of microtubules Induction of apoptosis	[5,91,97,98]
		Epothilones (e.g., Ixabepilone)		
**Intermediate Filaments**		Withaferin-A	Induction of apoptosis in vimentin-expressing cancer cells Inhibition of cellular proliferation Potent anti-angiogenic activity in vivo	[121,122,123]
		Silibinin	Inhibition of cell invasion and motility through the downregulation of vimentin and metalloproteinase-2	[124]
		Salinomycin	Downregulation of vimentin and upregulation of E-cadherin upon treatment	[125,126]
**Cytoskeletal-Associated Proteins**	**Integrins**	Abituzumab	Inhibition of pro-metastatic characteristics through enhancing detachment and inhibiting cancer cell adhesion	[127,128,129,130,131]
	**Tropomyosin**	Anti-Tropomyosin Drug TR100	Targets tropomyosin 5NM1/2 in the dissociation of actin from tropomyosin-mediated actin dynamics regulation to promote anti-cancer properties	[26,132,133]
		ATM-3507	Reduction in tumour growth and increased survival outcome	[133]
	**LIM kinase (LIMK)**	4-Pyridocarbazolone (LIMK1)	Mitotic microtubule disruption Inhibition of cofilin and actin dynamics Inhibition of cell motility and invasiveness in cancer	[134,135]
		6-Damnacanthal (LIMK1 and LIMK2)	Inhibition of cofilin and actin dynamics Inhibit cell motility and invasiveness in cancer	

**Table 2:** Examples of actin microfilament drug actions and cellular effects. Different actin microfilament drugs act to disrupt the actin dynamics and organisation in the cell. However, the importance of actin filaments in normal cell physiology also results in toxicity due to non-specificity (ATP, Adenosine Triphosphate).

Drug	Mechanistic Action	Cellular Effects	Toxicity	Ref.
**Cytochalasins**	Binds to F-actin at high affinity to inhibit microfilament nucleation and polymerisation	Inhibit tumour growth in cell lines of various cancer types—breast, lung and prostate Inhibit tumour growth and spontaneous metastasis in mouse	Congestion necrosis at the edge of the liver in rat Cardiac toxicity—change in stability of myofilament and reduce cardiac muscle contractibility	[101,102,103,104,105,106]
**Chaetoglobosin**	Prevents the nucleation of microfilament through the interaction with F-actin at the barb end of microfilaments	Inhibition of angiogenesis, cell growth and migration Activation of apoptosis	Induced necrosis of thymus and spleen as well as spermatocytes degeneration in mice Lethal at 2 mg/kg subcutaneous injection in Wistar rats	[107,108,109]
**Jasplakinolide**	Promotes the polymerisation and stability of microfilament by binding to F actin at multiple sites	Inhibition of growth in prostate carcinoma with the inhibition of actin cytoskeleton Induction of apoptosis	Cardiac toxicity due to its effect on several specific calcium and potassium ion channels in cardiomyocytes	[110,111,112,113]
**Latrunculins**	Inhibits microfilament polymerisation through interaction with G-actin monomer near the ATP binding site	Activate programmed cell death through the activation of caspase 3/7 pathway in gastric cancer Inhibition of tumour invasion in breast cancer	Induced chronic seizures with micro perfusion to the hippocampus of rat	[114,115,116,117]
**MKT-077**	Able to cross-link F-actin, leading to aberrant microfilaments	Cross linking promotes membrane ruffling and prevent Ras transformation of cells Inhibition of tumour growth and prolonged survival in vivo and in vitro	Renal toxicity, with hypomagnesemia and manageable toxicity such as peripheral oedema	[88,118,119,120]
